# Rickettsial Infection in Animals and Brazilian Spotted Fever Endemicity

**DOI:** 10.3201/eid1102.040656

**Published:** 2005-02

**Authors:** Luis A. Sangioni, Maurício C. Horta, Manoella C.B. Vianna, Solange M. Gennari, Rodrigo M. Soares, Márcio A.M. Galvão, Teresinha T.S. Schumaker, Fernando Ferreira, Odilon Vidotto, Marcelo B. Labruna

**Affiliations:** * University of São Paulo, São Paulo, São Paulo, Brazil;; † Centro Integrado de Ensino Superior – Campus Universitário, Campo Mourão, Paraná, Brazil;; ‡ Federal University of Ouro Preto, Ouro Preto, Minas Gerais, Brazil;; § Londrina State University, Londrina, Paraná, Brazil

**Keywords:** Brazilian spotted fever, Rickettsia rickettsii, Rickettsia parkeri, ticks, Amblyomma cajennense, surveillance, research

## Abstract

We compared the rickettsial infection status of *Amblyomma cajennense* ticks, humans, dogs, and horses in both Brazilian spotted fever (BSF)–endemic and –nonendemic areas in the state of São Paulo, Brazil. Most of the horses and few dogs from BSF-endemic areas had serologic titers against *Rickettsia rickettsii* antigens. In contrast, no dogs or horses from BSF-nonendemic areas had serologic titers against *R. rickettsii* antigens, although they were continually exposed to *A. cajennense* ticks. All human serum samples and ticks from both areas were negative by serologic assay and polymerase chain reaction, respectively. Our results indicate that surveys of horse serum are a useful method of BSF surveillance in areas where humans are exposed to *A. cajennense* ticks. In addition, we successfully performed experimental infection of *A. cajennense* ticks with *R. parkeri*.

## Abstract 2

Surveys of horse serum are a useful method of surveillance for Brazilian spotted fever in areas where humans are exposed to *Amblyomma cajennense* ticks.

Brazilian spotted fever (BSF) is an acute, febrile, tickborne disease caused by the bacterium *Rickettsia rickettsii*. The disease is transmitted by *Amblyomma* ticks and has been considered endemic in some areas of the states of São Paulo, Minas Gerais, Rio de Janeiro, and Espírito Santo (*1**–**7*). Although the tick species *Amblyomma aureolatum* is the main vector of BSF in few areas of the state of São Paulo (8, A. Pinter, unpub data), *A. cajennense* is the most common tick vector associated with the disease in Brazil (*9**–**11*).

*A. cajennense* is a common tick in rural areas of the state of São Paulo, where it is also the main tick species infesting humans (*12**,**13*). In contrast, BSF cases have been reported at only a few locations within the geographic range of this tick species (*14*). Although unreported cases may have occurred in other areas where BSF is not known to be endemic, this possibility is unlikely for such a highly lethal disease. Ecologic differences might be the main factor regulating the occurrence of *R. rickettsii* among ticks and, consequently, the occurrence of the disease.

The infection rate by *R. rickettsii* within a tick population can be diminished or even suppressed when a second *Rickettsia* species infects most of the members of that tick population (*15**,**16*). Thus, we hypothesize that the absence of human cases of BSF in some areas of the state of São Paulo (where human parasitism by *A. cajennense* is intense) is related to the presence of other, less pathogenic *Rickettsia* species infecting *A. cajennense* tick populations. In this regard, our study evaluated the rickettsial infection status of *A. cajennense* populations from both BSF-endemic and -nonendemic areas in the state of São Paulo. We also serologically evaluated humans and domestic animals from these BSF-nonendemic areas to compare it to a recent evaluation that we performed in BSF-endemic areas (*17*).

## Materials and Methods

### Study Area

The study was conducted on 6 farms in the state of São Paulo. Three of these farms (farms 1, 2, and 3) were considered endemic for BSF because of the recent occurrence of several laboratory-confirmed human cases of the disease among residents (*4**,**14*). These farms were the same ones evaluated in a study of Horta et al. (*17*). The remaining 3 farms (4, 5, and 6) were considered nonendemic for BSF because they had never had human cases of this disease. However, *A. cajennense* ticks were abundant there, and human infestation by this tick was a normal finding year-round among farm residents. Farms 1 (22°44´19´´S, 46°55´27´´W), 2 (22°47´03´´S, 46°54´10´´W) and 3 (22°41´14´´S, 46°53´17´´W) were located in the Pedreira Municipality whereas farms 4 (23°23´15´´S, 47°26´14´´W), 5 (23°36´43´´S, 46°57´29´´W), and 6 (21°57´07´´S, 47°27´05´´W) were located in Porto Feliz, Cotia, and Pirassununga Municipalities, respectively.

In all 6 farms, human occupations were basically divided between livestock-raising activities for men and household activities for women and children. Nevertheless, children spent substantial time in outdoor activities. All 6 farms had horses grazing on mixed overgrowth pastures, interspersed with remote forest areas. However, the major ecologic difference was large populations of free-living capybaras that inhabited livestock pastures on farms 1, 2, and 3 and the absence of this animal from horse pastures on farms 4, 5, and 6. All farms, except farm 4, had free-roaming dogs with free access to pasture and forest areas. Recent studies on ticks collected on the pastures and on horses and dogs from these 6 farms allowed the tick species *A. cajennense* and *Dermacentor nitens* to be identified on the 6 farms. In addition, the capybara tick, *A. cooperi*, was present on farms 1, 2, and 3 but absent in the pastures of farms 4, 5, and 6 (*13**,**17**–**19*). Human infestation by *Amblyomma* ticks was frequent on all the farms.

### Ticks

From December 2000 to March 2001, free-living *A. cajennense* adult ticks were collected from horse pastures of the 6 farms by dragging and by using CO_2_ traps. Totals of ticks collected from the farms are as follows: farm l (244), farm 2 (353), farm 3 (213), farm 4 (222), farm 5 (206), and farm 6 (230). All ticks were brought alive to the laboratory, where their surfaces were disinfected by immersion in 70% alcohol for 10 min followed by washing in sterile water; they were then individually tested by the hemolymph test (*20*). Briefly, a drop of hemolymph of each tick was dried on a glass slide and stained by the Gimenez method (*21*). Thereafter, ticks were frozen at –80°C until processed for DNA extraction.

### DNA Extraction

All ticks were processed individually for DNA extraction. Each tick was cut into 2 symmetric halves through its median axis. One half was returned to the –80°C freezer for further studies, and the other half was used for DNA extraction according to a modification of a previously described protocol (*22*). For this purpose, each tick half was placed in a 1.5-mL microtube containing 150 µL of TE buffer (Tris HCl 10 mmol/L, EDTA 1 mmol/L, pH 7.4) and homogenized by using a sterile micropestle. Microtubes containing the homogenized, triturated ticks were then vortexed vigorously. Next, 450 µL of guanidine thiocyanate (5 mol/L) were added to the tube, which was vortexed again and incubated for 10 min at room temperature with short vortexing every 2 min. Thereafter, 100 µL of chloroform was added to the tube, which was inverted several times and left resting for 2 min. The tube was centrifuged at 12,000 x *g* for 5 min to separate the aqueous phase, which was transferred to a clean 1.5-mL microtube. Next, 600 µL of isopropanol was added to the aqueous phase (400 mL), which was homogenized by inverting the tube several times and then incubated at –20°C for 2 to 18 h. Thereafter, the tube was centrifuged at 12,000 x *g* for 15 min; the supernatant was discarded, and the pellet was dried at room temperature and then resuspended with 30 µL of buffer TE. Finally, the microtubes were incubated at 56°C for 15 min to facilitate DNA homogenization and then stored at –20°C until tested by polymerase chain reaction (PCR).

### PCR

Five microliters of the extracted DNA from tick specimen was used as template for amplification of fragments of the rickettsial *gltA* (citrate synthase gene) and 17-kDa protein gene. A 381 – bp portion of the *Rickettsia gltA* gene was targeted from each extracted tick DNA by using primers RpCS.877 and RpCS.1258n (*23*), and a 434 – bp portion of the *Rickettsia* genus-specific 17-kDa protein gene was targeted as previously described (*24*). Ten microliters of the PCR product underwent electrophoresis in 1.5% agarose gel, stained with ethidium bromide, and examined with UV transillumination. For the 10 individual ticks that were tested by PCR, a negative control (5 µL of water) and positive control (5 µL of DNA extracted from an *A. cajennense* tick experimentally infected with *R. parkeri*) were included. Procedures to obtain *R. parkeri* experimentally infected ticks are described below. PCR results were statistically analyzed by the program @Risk Software – Risk Analysis Add-in for Microsoft Excel (Palisade Corporation, Newfield, NY, USA), which adopted Monte Carlo techniques to determine the confidence level of the prevalence of ticks infected by *Rickettsia* in each tick population (farm), considering α = 0.05.

### *R. parkeri* Experimentally Infected Ticks

Purified *R. parkeri* organisms (Maculatum strain) were obtained by the renografin purification method from infected Vero cells (*25*). The resultant purified rickettsiae were resuspended in sucrose-phosphate-glutamic acid buffer and stored frozen at –80°C until tick infection. Seventy adult specimens of *A. cajennense* were obtained from the third generation of our laboratory colony at the University of São Paulo. This colony was established 15 months earlier from engorged females collected on horses on farm 6 of the present study. Adult ticks had their dorsum attached to double-face adhesive tape, which was taped onto petri dishes. Purified stock of *R. parkeri* was thawed at room temperature, and each tick was injected by using a 28-gauge microfine insulin needle. Under a stereoscopic microscope, a small drop (≈2 µL) of *R. parkeri* suspension was injected into the coelom of the tick, through the articulation of coxa IV with trochanter IV, in each of 50 adult ticks. A control group of 20 ticks were injected by the same procedure with phosphate-buffered saline (PBS). Ticks were removed from the adhesive tape and held in an incubator at 35°C and relative humidity >95% for 5 days. Ticks were tested by the hemolymph test as described above, at days 3 and 5 after infection. Thereafter, ticks were frozen at –80°C. DNA of hemolymph-positive ticks was extracted, as described above, to be used as positive control for our PCR assays. A sample of 10 PBS-injected ticks were also tested by the PCR method described above.

### Domestic Animals and Humans

During our visit to farms 1 to 6, blood samples were collected from 100% of the dogs and horses on each farm and ≈90% of the resident humans. Blood samples were transported at room temperature to the laboratory, where samples were centrifuged (1,500 x *g*, 10 min), and the sera were aliquoted into labeled microtubes and stored at –20°C until tested by the indirect immunofluorescence assay (IFA) with *R. rickettsii* antigen, as described (*17*). The serologic results of farms 1 to 3 have been reported by Horta et al. (*17*) and will be compared with our results for farms 4 to 6. Collection of animal and human samples was previously approved by ethical principles in animal and human research of the University of São Paulo.

## Results

### Field Ticks

A total of 1,468 *A. cajennense* adult ticks (810 from disease-endemic and 658 from disease-nonendemic areas) were tested by the hemolymph test. They were all negative. These same ticks were also negative by the PCR protocols targeting the rickettsial genes *gltA* and 17-kDa protein. In all PCR assays, DNA of *A. cajennense* ticks experimentally infected with *R. parkeri* (positive controls) yielded the expected bands whereas no bands were obtained for the negative controls.

Our results, after being analyzed by the Monte Carlo techniques, are that on farm 5, where 206 ticks (our smallest sample) were tested, the prevalence of *A. cajennense* ticks infected by *Rickettsia* was at most 1.43% (upper limit of 95% confidence interval). If the prevalence was higher than this value, infection in at least 1 tick would have been detected by our PCR. Similarly, in farm 2, where 353 ticks were tested (our largest sample), the prevalence of ticks infected by *Rickettsia* was at most 0.8% (upper limit of 95% confidence interval). Overall, these analyses indicated that the prevalence of rickettsial infection on the 6 farms was no more than 0.8%–1.43%. As we used *Rickettsia* genus specific primers in the PCR, this infection could be due to any *Rickettsia* species.

### Ticks Experimentally Infected with *R. parkeri*

Of 50 ticks infected with *R. parkeri*,10 (20%) showed typical *Rickettsia*-like organisms within the hemocytes 3 days after injection. On day 5, the number of ticks showing typical *Rickettsia*-like organisms in their hemocytes increased to 28 (56%). None of the 20 ticks injected with PBS showed *Rickettsia*-like organisms in their hemolymph 3 or 5 days after injection. All 28 hemolymph-positive ticks yielded expected bands in both PCR protocols (*gltA* and 17-kDa protein) whereas no PBS-injected ticks yielded amplified DNA bands.

### Serologic Assays

Serum samples were collected from horses, dogs, and humans from the 6 farms, as shown in the Table. From the BSF-nonendemic areas (farms 4–6), no sample from a dog, horse, or human reacted positively with *R. rickettsii* antigens. The serologic results for the BSF-endemic areas (farms 1–3) were reported by Horta et al. (*17*). The proportion of horses that reacted positively with *R. rickettsii* antigens (titer >64) varied from 57.1% to 80%; for dogs, these proportions varied from 0% to 66.7%. Like farms 4–6, no human serum sample from farms 1 to 3 reacted positively with *R. rickettsii* antigens.

**Table:** Results of indirect immunofluorescence assay for antibodies to *Rickettsia rickettsii* in humans and domestic animals from 3 BSF-endemic areas (farms 1–3)* and 3 BSF-nonendemic areas (farms 4–6), São Paulo, Brazil†

Source	Reactive sera‡/total sera tested (% reactive)					
	Farm 1	Farm 2	Farm 3	Farm 4	Farm 5	Farm 6
Humans	0/20 (0)	0/21 (0)	0/9 (0)	0/4 (0)	0/2 (0)	0/10 (0)
Horses	9/10 (90)	4/7 (57.1)	4/5 (80)	0/16 (0)	0/10 (0)	0/21 (0)
Dogs	1/4 (25)	4/6 (66.7)	0/6 (0)	No dogs	0/4 (0)	0/1 (0)

*Data from Horta et al. (*17*). †BSF, Brazilian spotted fever. ‡Sera showing titers >64 for *R. rickettsii* antigen.

## Discussion

Our study evaluated *A. cajennense* ticks in BSF-endemic and -nonendemic areas in the state of São Paulo. In addition, we serologically evaluated domestic animals and humans from BSF-nonendemic areas and compared the results with a previous serologic evaluation in BSF-endemic areas (*17*). Our results for the nonendemic areas showed no evidence of a pathogenic *Rickettsia* species circulating in *A. cajennense* ticks in farms 4 to 6, since all animals, humans, and ticks were negative. In contrast, Horta et al. (*17*) showed serologic evidence of *R. rickettsii* infection by cross-absorption and IFA analyses in most of the horses and some dogs in the 3 BSF-endemic areas (farms 1–3), a finding that is supported by the recent occurrence of human BSF cases in those farms. The serologic reactivity of horses, dogs, and humans to *R. rickettsii* antigen in BSF-endemic areas where *A. cajennense* is the main vector is characterized by a high frequency of serologically positive horses, followed by a lower frequency in dogs, and an even lower frequency or absence of serologically positive humans (*17*). This pattern has been observed in several BSF-endemic areas in which *A. cajennense* has been incriminated as the vector (*3**,**17**,**26**,**27*). The absence of serologic reactivity among the human residents whom we tested is supported by their lack of history of the disease; previous cases reported in this area were lethal or if not, the survivors do not live in the BSF-endemic area anymore.

Horses are one of the most important hosts for *A. cajennense* in the state of São Paulo; both immature and adult ticks will successfully feed on this animal (*18*). This fact makes the horse an excellent sentinel for BSF surveillance. Once the *A. cajennense* population increases in an area, parasitic stages will have a greater chance to successfully feed on other host species, including dogs and humans. As dogs are naturally infested with ticks more frequently than humans, they are also a good sentinel for BSF surveillance. Results of our study support this statement because our serologic survey of horses and dogs from 3 areas, where no BSF case has been reported, indicated that neither *R. rickettsii* nor a closely related species circulated in the local *A. cajennense* ticks. Thus, we recommend surveys of horse sera as a useful method for BSF surveillance in areas where humans are exposed to *A. cajennense* ticks. This procedure would allow potentially BSF-endemic areas to be identified before human cases occur.

We failed to detect any rickettsial DNA in the field-collected *A. cajennense* ticks. Although this result is supported by the serologic results in the BSF-nonendemic areas, it was not expected for the BSF-endemic areas, where infection by *R. rickettsii* in horses and dogs has been indirectly proven by serologic cross-absorption methods (*17*). Finding *R. rickettsii*-infected ticks in spotted fever–endemic areas can be difficult. In North Carolina, a U.S. state with a high incidence of Rocky Mountain spotted fever (caused by *R. rickettsii*), only 1 of 2,123 *Dermacentor variabilis* ticks studied was infected by *R. rickettsii* (*15*). Thus, further studies in São Paulo should encompass a much larger number of *A. cajennense* ticks.

The major ecologic differences between the BSF-endemic and -nonendemic areas of our study were the presence of capybaras and their main tick species (*A. cooperi*), found solely in the BSF-endemic areas. In a recent survey of rickettsiae in *A. cooperi* ticks collected on farms 1, 2, and 3 (*19*), 2 rickettsiae were isolated from these ticks: *R. bellii* and a *Rickettsia* species (strain COOPERI) closely related to *R. parkeri* and *R. africae*. Similar to the present study, no *R. rickettsii* was found infecting *A. cooperi* ticks.

Burgdorfer et al. (*16*) found that high infection rates (up to 80%) by a less pathogenic rickettsia were the limiting factor for establishing *R. rickettsii* in the *D. andersoni* tick population of the east side of the Bitterroot Valley in Montana, USA. On the west side of this valley, where 8%–16% of the ticks were infected by the less pathogenic rickettsia, disease caused by *R. rickettsii* was endemic. Based on these observations, the results of our study suggest that unknown factors other than the presence of different *Rickettsia* species are responsible for the absence of a pathogenic spotted fever group rickettsia's infection of populations of *A. cajennense* populations in farms 4, 5, and to 6 (BSF-nonendemic areas).

In a recent study performed in our laboratory (A. Pinter and M.B. Labruna, unpub. data) *R. rickettsii* was detected in 6 (0.89%) of 669 *A. aureolatum* adult ticks by using the same PCR protocols as the present study. These ticks were collected in a different BSF-endemic area, in which *A. aureolatum* is the main vector of the disease. As our results showed that the highest predictable infection rate of *R. rickettsii* in the *A. cajennense* population of farm 3 (where 353 ticks were tested) was 0.8%, we might have found a *R. rickettsii*–infected *A. cajennense* tick if we had tested a larger sample of ticks from that farm. Even though recent studies have failed to detect or isolate *R. rickettsii* from *A. cajennense* ticks in Brazil, earlier studies detected it efficiently in the states of São Paulo (*28*) and Minas Gerais (*9**,**10*), as well as in Colombia (*29*), Mexico (*30*), and Panama (*31*).

Our study showed that *R. parkeri* could experimentally infect *A. cajennense* ticks. A previous, more extensive, study showed that *A. americanum* ticks experimentally infected with *R. parkeri* were able to maintain this infection for 2 generations and were able to transmit it to guinea pigs through tick feeding (*32*). Natural infection of ticks by this agent has been reported in *A. maculatum* (*33*) and *A. triste* (*34*). The *Rickettsia* species (strain COOPERI), found to be infecting *A. cooperi* ticks in São Paulo state (*19*), seems to be another strain of *R. parkeri* or a closely related species. These results show that *R. parkeri* can infect different *Amblyomma* species under experimental or natural conditions. The potential role of *A. cajennense* to transmit *R. parkeri* in nature requires further investigation, especially since *R. parkeri* was recently shown to be pathogenic for humans (*35*).

## References

[1] Gonçalves AJR, Lopes PFA, Melo JCP, Pereira AA, Pinto AMM, Lazera MS, Rickettsioses. A propósito de quatro casos diagnosticados no Rio de Janeiro de febre amculosa barsileira. Folha Med. 1981;82:127–34.

[2] Sexton DJ, Muniz M, Corey GR, Breitschwerdt EB, Hegarty BC, Dumler S, Brazilian spotted fever in Espirito Santo, Brazil: description of a focus of infection in a new endemic region. Am J Trop Med Hyg. 1993;49:222–6. 8357085 10.4269/ajtmh.1993.49.222

[3] Lemos ER, Machado RD, Coura JR. Rocky Mountain spotted fever in an endemic area in Minas Gerais, Brazil. Mem Inst Oswaldo Cruz. 1994;89:497–501. 10.1590/S0074-02761994000400001 8524052

[4] Lemos ER, Alvarenga FB, Cintra ML, Ramos MC, Paddock CD, Ferebee TL, Spotted fever in Brazil: a seroepidemiological study and description of clinical cases in an endemic area in the state of Sao Paulo. Am J Trop Med Hyg. 2001;65:329–34. 11693878 10.4269/ajtmh.2001.65.329

[5] Lemos ER, Rozental T, Villela CL. Brazilian spotted fever: description of a fatal clinical case in the State of Rio de Janeiro. Rev Soc Bras Med Trop. 2002;35:523–5. 10.1590/S0037-86822002000500017 12621675

[6] Galvao MA, Lamounier JA, Bonomo E, Tropia MS, Rezende EG, Calic SB, Rickettsioses emergentes e reemergentes numa região endêmica do estado de Minas Gerais, Brazil. Cad Saude Publica. 2002;18:1593–7. 10.1590/S0102-311X2002000600013 12488886

[7] Galvao MA, Calic SB, Chamone CB, Mafra SCL, Cesarino Filho G, Olano JP, Spotted fever rickettsiosis in Coronel Fabriciano, Minas Gerais State. Rev Soc Bras Med Trop. 2003;36:479–81. 10.1590/S0037-86822003000400008 12937725

[8] Gomes LS. Thypho exanthematico de São Paulo. Brasil Médico. 1933;17(52):919–21.

[9] Moreira JA, Magalhães O. Thypho exanthematico em Minas Gerais. Brasil Médico. 1935;44:465–70.

[10] Dias E, Martins A, Ribeiro DJ. Thypho exanthematico no Oeste de Minas Gerais. Brasil Médico. 1937;51:651–5.

[11] Lima VLC, Figueiredo AC, Pignatti MG, Modolo M. Febre maculosa no município de Pedreira, Estado de São Paulo, Brasil: Relação entre ocorrência de casos e parasitismo humano por ixodídeos. Rev Soc Bras Med Trop. 1995;28:135–7. 7716327 10.1590/s0037-86821995000200010

[12] Guimarães JH, Tucci EC, Barros-Battesti DM. Ectoparasitos de importância veterinária. São Paulo: Editora Plêiade; 2001.

[13] Labruna MB, Kerber CE, Ferreira F, Faccini JLH, De Waal DT, Gennari SM. Risk factors to tick infestations and their occurrence on horses in the State of São Paulo, Brazil. Vet Parasitol. 2001;97:1–14. 10.1016/S0304-4017(01)00387-9 11337122

[14] Lima VL, Souza SS, Souza CE, Vilela MF, Papaiordanou PM, Del Guercio VM, Situação da febre maculosa na região administrativa de Campinas, São Paulo, Brasil. Cad Saude Publica. 2003;19:331–4. 10.1590/S0102-311X2003000100038 12700815

[15] Burgdorfer W. Ecological and epidemiological considerations of Rocky Mountain spotted fever and scrub typhus. In: Walker DH, editor. Biology of rickettsial diseases. Vol. 1. Boca Raton (FL): CRC Inc.; 1988. p. 33–50.

[16] Burgdorfer W, Hayes SF, Mavros AJ. Nonpathogenic rickettsiae in *Dermacentor andersoni*: a limiting factor for the distribution of *Rickettsia rickettsii*. In: Burgdorfer W, Anacker RL, editors. Rickettsiae and rickettsial diseases. New York: Academic Press; 1981. p.585–94.

[17] Horta MC, Labruna MB, Sangioni LA, Vianna MCB, Gennari MS, Galvão MAM, Prevalence of antibodies to spotted fever group rickettsiae in humans and domestic animals in a Brazilian Spotted fever endemic area in the state of São Paulo, Brazil: serological evidence for infection by *Rickettsia rickettsii* and another spotted fever group rickettsia. Am J Trop Med Hyg. 2004;71:93–7. 15238696

[18] Labruna MB, Kasai N, Ferreira F, Faccini JLH, Gennari SM. Seasonal dynamics of ticks (Acari: Ixodidae) on horses in the state of São Paulo, Brazil. Vet Parasitol. 2002;105:65–72. 10.1016/S0304-4017(01)00649-5 11879967

[19] Labruna MB, Whitworth T, Horta MC, Bouyer DH, McBride JW, Pinter A, *Rickettsia* species infecting *Amblyomma cooperi* ticks from an endemic area for Brazilian spotted fever in the state of São Paulo, Brazil. J Clin Microbiol. 2004;42:90–8. 10.1128/JCM.42.1.90-98.2004 14715737 PMC321730

[20] Burgdorfer W. The hemolymph test. Am J Trop Med Hyg. 1970;19:1010–4. 4992724

[21] Gimémez DF. Staining rickettsiae in yolk-sac cultures. Stain Technol. 1964;39:135–40. 14157454 10.3109/10520296409061219

[22] Chomkzynski P. A reagent for the single-step simultaneous isolation of RNA, DNA and proteins from cell and tissue samples. Biotechniques. 1993;15:532–7. 7692896

[23] Regnery RL, Spruill CL, Plikaytis BD. Genotypic identification of rickettsiae and estimation of intraspecies sequence divergence for portions of two rickettsial genes. J Bacteriol. 1991;173:1576–89. 1671856 10.1128/jb.173.5.1576-1589.1991 PMC207306

[24] Webb L, Carl M, Malloy DC, Dasch GA, Azad AF. Detection of murine typhus infection in fleas by using the polymerase chain reaction. J Clin Microbiol. 1990;28:530–4. 2108995 10.1128/jcm.28.3.530-534.1990 PMC269657

[25] Hanson BA, Wisseman CL Jr, Waddell A, Silverman DJ. Some characteristics of heavy and light bands of *Rickettsia prowazekii* on Renografin gradients. Infect Immun. 1981;34:596–604. 6796519 10.1128/iai.34.2.596-604.1981 PMC350908

[26] Lemos ERS, Machado RD, Coura JR, Guimarães MAA, Chagas N. Epidemiological aspects of the Brazilian spotted fever: serological survey of dogs and horses in an endemic area in the state of São Paulo, Brazil. Rev Inst Med Trop Sao Paulo. 1996;38:427–30. 10.1590/S0036-46651996000600007 9293089

[27] Galvão MAM. Febre maculosa em Minas Gerais: um estudo sobre a distribuição da doença no Estado e seu comportamento em área de foco peri-urbano [Doctoral thesis]. Belo Horizonte: Faculdade de Medicina da UFMG; 1996. p. 114.

[28] Vallejo-Freire A. Spotted fever in Mexico. Memórias doInstituto Butantan.1946;19:159–80.

[29] Patino-Camargo L. Nuevas observaciones sobre un tercer foco de fiebre petequial (maculosa) en el hemisferio americano. Bol Oficina Sanit Panam. 1941;20:1112–4.

[30] Bustamante ME, Varela G. Estudios de fiebre manchada en Mexico. Hallazgo del *Amblyomma cajennense* naturalmente infectado, en Veracruz. Rev Inst Salubr Enferm Trop. 1946;7:75–8.

[31] Rodaniche EC. Natural infection of the tick *Amblyomma cajennense* with *Rickettsia rickettsii* in Panama. Am J Trop Med Hyg. 1953;2:696–9. 13065638 10.4269/ajtmh.1953.2.696

[32] Goddard J. Experimental infection of lone star ticks, *Amblyomma americanum* (L.), with *Rickettsia parkeri* and exposure of guinea pigs to the agent. J Med Entomol. 2003;40:686–9. 10.1603/0022-2585-40.5.686 14596284

[33] Lackman D, Parker R, Geloff R. Serological characteristics of a pathogenic rickettsia occuring in *Amblyomma maculatum.* Public Health Rep. 1949;64:1342–9. 10.2307/4587134 15398100

[34] Venzal JM, Portillo A, Estrada-Peña A, Castro O, Cabrera PA, Oteo JA. *Rickettsia parkeri* in *Amblyomma triste* from Uruguay. Emerg Infect Dis. 2004;10:1493–5. 15496258 10.3201/eid1008.030999 PMC3320401

[35] Paddock CD, Sumner JW, Comer JA, Zaki SR, Goldsmith CS, Goddard J, *Rickettsia parkeri*: a newly recognized cause of spotted fever rickettsiosis in the United States. Clin Infect Dis. 2004;38:805–11. 10.1086/381894 14999622

